# Supplementary material for: Development of Data Transfer Ethics Framework (daTEF): A participatory approach to delivering evidence-based guidelines for healthcare data transfer
Source: PLoS One. 2025 Nov 10;20(11):e0336389. doi: 10.1371/journal.pone.0336389 (PMC12599928; doi:10.1371/journal.pone.0336389)
Supplement: S1 File — (PDF) [file pone.0336389.s001.pdf]

Supplementary File

Guideline for Data Transfer in Research: A Nepalese Perspective

**Guideline Statement**	
**Purpose**	The purpose of this guideline is to provide recommendations for researchers and institutions in Nepal to overcome data-transfer-related challenges and promote secure, ethical, and efficient data-sharing practices, ultimately advancing research and collaboration in the country.
**Scope**	This guideline is specifically tailored to address data transfer challenges in the context of research, with a focus on the unique challenges and opportunities faced by researchers working at non-profit and non-governmental research institutions in Nepal.
**Guideline**	

Guideline for Data Transfer in Research

The guideline serves as a framework for addressing data transfer challenges in research, with a specific focus on the context of Nepalese researchers working at non-profit and non-governmental research institutions in Nepal. This guideline is made to give data-transfer-based contextual challenges and associated solutions that may help researchers and institutions follow the rules about sharing data in research. This guideline aims to explain what the rules mean and why the discussion for it is important. Each section within the guideline explains a major pillar of contextual challenges and the following subheadings explain the major challenges in point and associated recommendations that were collected from the researchers working at non-profit and non-governmental research institutions in Nepal.

Section 1: Standardized Guidelines and Legal Frameworks

I. The lack of standardized guidelines from the government or associated institutes for data transfer

a. Identified Challenges:

1. Lack of framework provided by/from the government.
2. Lack of government departments and laws that protect intellectual property (IP).
3. Lack of an independent department that oversees research.
4. Lack of national/public depository.

b. Recommendations and Solutions:

1. Government/Institution-led Standardization:

The government should appoint individuals with scientific backgrounds to standardize the framework for data sharing. These experts will help prevent valuable data from being misused or shared inappropriately, especially if it's considered a national treasure.

2. Government/Institution-led Legal Protection and Oversight:

The government/institutions must establish respective departments/committees and associated laws dedicated to safeguarding intellectual property rights (IPR). Updates to these laws should reflect global best practices, giving Nepali researchers confidence in the protection of their research and patents.

3. Data Depository:

The government/institutions should establish a comprehensive, open national/public data depository to archive and regulate all research findings in Nepal. This should include healthcare and biological data, ensuring preservation for future research.

4. Government-led Independent Regulatory Body:

The government should create an independent department to oversee research approvals and the sharing of results and samples. This regulatory body will ensure the correspondence of proposed research and work.

Section 2: Awareness and Training among Researchers about the Data Transfer Process

II. Inadequate awareness and training in data sharing

a. Identified Challenges:

1. Lack of knowledge of data sharing among Researchers about the Data Transfer Process.

2. Requirement for specialized data sharing training among Researchers about the Data Transfer Process.

b. Recommendations and Solutions:

1. Government/Institution-Led Training:

The government/institution should take responsibility for training new students, trainees and researchers in data-sharing best practices. This should be a mandatory part of the research ecosystem.

2. Educational Reform:

An overhaul of the education system is necessary to include a curriculum on intellectual property (IP), patents, authorship, and data ownership. Or at least, government/institution-led short courses or learning modules should be devised on a case-by-case basis.

Section 3: Issues Related to Data Sharing

III. Problems related to data sharing

a. Identified Challenges:

1. Misuse, manipulation, and theft of data by collaborator and/or third party.

2. Conflicts between parties (collaborator and/or third party).

3. Uncertainty about data usage, including plagiarism and theft.

b. Recommendations and Solutions:

1. Enforce Strict Data Protection Laws:

The government/institutions should implement and enforce stringent rules and laws to protect researchers' data. This includes strong Intellectual Property Rights (IPR) protection to prevent unauthorized access, theft, or misuse of research data.

2. Clear Agreements:

Organizations and collaborators should have clear, written agreements including the material transfer agreement (MTA). These agreements should define data ownership, authorship, the role of the funder and the percentage of patents in a collaborative project.

3. Ownership and Usage Agreements:

Both parties should sign an agreement at the outset of a project, explicitly stating data ownership and usage rights. This should help prevent conflicts in the future.

4. Strict Legal Measures:

Strict laws should be in place to address issues of plagiarism and data theft, including legal action and penalties for those found in violation.

Section 4: Sample Transfer Challenges

IV. Problems related to biological sample transfer.

a. Identified Challenges:

1. Scientific samples from foreign countries aren't allowed in Nepal, which creates a one-way pass of biological samples from Nepal to international institutions.

2. Challenges related to temperature maintenance during sample transfer.

3. Excessive paperwork delays sample transfer.

4. Lack of a framework for sample transfer.

5. Limited courier options.

b. Recommendations and Solutions:

1. *Sample Import Regulations:*

The government should update and enforce material agreements to facilitate the import of scientific samples from countries with standard documentation. This allows researchers to access resources not available in Nepal. If there are old/no sample import guidelines, they should be updated and/or created.

2. *Temperature Maintenance Facilities:*

The government/institutions should expand options for material transfer with methods that maintain stable temperatures. Develop facilities for temperature maintenance when transferring or receiving samples.

3. *Streamlined Protocols:*

Develop a standardized, convenient protocol for sample transfer that can be carried out online, reducing unnecessary paperwork and delays.

4. *Framework for Sample Transfer:*

The government should create a standard framework that local and foreign researchers can conveniently use to collaborate and transfer samples.

5. *Flexible Courier Options:*

Allow researchers to choose their preferred courier services, offering faster and more reliable means for sample transfer.

Section 5: Conclusion

A robust data transfer process is crucial for research collaboration and scientific advancement. By following this comprehensive guideline, researchers, institutions, and government bodies can overcome the identified challenges and promote secure, ethical, and efficient data-sharing practices in Nepal. This will contribute to the growth and success of the research landscape in the country.

**Accountabilities**	
**Responsible Officer**	Title of the senior executive having the greatest overall responsibility for the subject area that this Guideline relates to.
**Contact Officer**	Title of the person responsible for the day-to-day management of this Guideline and who will assist with queries.
**Supporting Information**	
**Legislative Compliance**	This Guideline supports the Institution's compliance with the following legislation:<br>List the full titles and URLs of any applicable State or Federal legislation, including any relevant pinpoint references. If relevant, list any statutes/regulations that this Guideline relates to. Any variation to this Guideline must remain consistent with the parent statute or regulation.<br>If none, insert "Nil".<br>Contact the Institutional Compliance Manager for further advice when completing this section.
**Parent Document (Policy and Procedure)**	Include the title and URLs of any Policy and Procedure that this Guideline supports. If none, insert 'Nil'.
**Supporting Documents**	Include titles and URLs of templates or forms that directly support this Guideline. If none, insert 'Nil".
**Superseded Documents**	List all documents superseded by this Guideline. If none, insert 'Nil'.
**File Number**	[For Governance Use]

**Revision History**				
**Version**	**Approved by**	**Approval date**	**Effective date**	**Sections modified**
#.#	[to be completed]	[to be completed]	[to be completed]	[to be completed]
