# Supplementary material for: Development of Data Transfer Ethics Framework (daTEF): A participatory approach to delivering evidence-based guidelines for healthcare data transfer
Source: PLoS One. 2025 Nov 10;20(11):e0336389. doi: 10.1371/journal.pone.0336389 (PMC12599928; doi:10.1371/journal.pone.0336389)
Supplement: S2 File — (PDF) [file pone.0336389.s002.pdf]

Characterizing the challenges experienced in health care data exchange between Nepalese and international research team to develop a local data transfer framework

Interview/Survey Consent Form.

You are being invited to participate in a research study titled:

Characterizing the challenges experienced in health care data exchange between Nepalese and international research team to develop a local data transfer framework.

The purpose of this research study:

To develop a guideline for addressing syndemic (the co-occurrence of more than one epidemic) challenges observed during the implementation and execution of healthcare research that involves healthcare data transfer outside of Nepal.

Investigation Team:

Principal Investigator: Anurag Adhikari, PhD

Co-Investigator: Ms. Minu Singh

Co-Investigator: Mr. Gopiram Syangtan

Name of Research institution: Department of Infection and Immunology, Kathmandu Research Institute for Biological Sciences (KRIBS)

Participant Consent

I have read and understood the participant information sheet.

☐ Yes
☐ No

I have been given the opportunity to ask questions about the study.

☐ Yes
☐ No

I agree to participate in the study. Taking part in the project will include being interviewed and recorded (audio or video).

☐ Yes
☐ No

I understand that my participation is voluntary; I can withdraw from the study at any time, and I do not have to give any reasons for why I no longer want to involve in this study.

- ☐ Yes
- ☐ No

<u>Use of the information I provide for this study only</u>

I understand my personal details/information will not be disclosed other than research team members.

- ☐ Yes
- ☐ No

I understand that my words may be quoted anonymously in publications, reports, web pages, and other research outputs.

- ☐ Yes
- ☐ No

<u>Use of the information I provide beyond this project.</u>

I agree for the data I provide to be archived.

- ☐ Yes
- ☐ No

I understand that other researchers within KRIBS or outside KRIBS will have access to this data only if they agree to preserve the confidentiality of the information as requested in this form.

- ☐ Yes
- ☐ No

I understand that other researchers within KRIBS or other than KRIBS may use my words in publications, reports, web pages, and other research outputs, only if they agree to preserve the confidentiality of the information as requested in this form.

- ☐ Yes
- ☐ No

Name of Participant:

Signature:

Date:

Characterizing the challenges experienced in health care data exchange between Nepalese and international research team to develop a local data transfer framework

अन्तर्वार्ता/सर्वेक्षण सहमति फारम

तपाईंलाई यस शीर्षकको अनुसन्धानमा भाग लिनको लागि आमन्त्रित गरिएको छः

डाटा ट्रान्सफर फ्रेमवर्क विकास गर्न नेपाली र अन्तर्राष्ट्रिय अनुसन्धान टोलीबीच स्वास्थ्य सेवा डाटा आदानप्रदानमा अनुभव भएका चुनौती चित्रण

यस अनुसन्धानको उद्देश्य:

हाम्रो डाटाहरु ट्रान्सफर गरि स्वास्थ्य बिधाका अनुसन्धानहरुलाई प्रभावकारी रुपमा कार्यान्वयन गर्न र कार्यान्वयनको क्रममा देखिएका समस्य र चुनौतीहरुलाई समाधान गर्नका लागि निर्देशिका निर्माण गर्न

अन्वेषकहरु

प्रमुख अन्वेषक: अनुराग अधिकारी
सह-अन्वेषक: मिनु सिंह
सह-अन्वेषक: गोपीराम स्याङ्तान

अनुसन्धान गर्ने संस्थाको नाम:

डिपर्ट्मेन्ट अफ इन्फेक्सन एण्ड इम्युनोलोजी

काठमाडौं रिसर्च ईन्स्टिच्यु फर बायोलोजीकल साइन्सेस (कृबस)

बायोमेडिकम रिसर्च क्याम्पस भवन सप्तखेल-९,

बालकुमारी, च्यासल रोड

ललितपुर ४४७००, बागमती प्रदेश, नेपाल

सहभागी सहमति सम्बन्धमा

मैले सहभागी बारेमा जानकारी पत्र पढेको र बुझेको छ |

- ☐ छ
- ☐ छैन

मैले यस अनुसन्धानको बारेमा मलाई प्रश्न सोध्ने मौका दिइएको छ।

- ☐ छ
- ☐ छैन

म यस अनुसन्धान परियोजनामा सहभागी हुन सहमत छु । यस अनुसन्धान परियोजनामा सहभागी हुँदा मेरो अन्तर्वार्ताको (रेकर्डिङ अडियो वा भिडियो) समावेश हुनेछ?

- ☐ छ
- ☐ छैन

यस अनुसन्धानमा मेरो सहभागीता पुर्ण रुपमा भोलिन्टियर हो र म कुनै पनि समयमा यस अनुसन्धानबाट अलग हुन सक्छु त्यसको लागि कुनै पनि कारण दिनु पर्ने छैन?

- ☐ छ
- ☐ छैन

मैले यो सूचना यस अनुसन्धानको लागि मात्र प्रयोग गर्न दिइएको छ।

मेरो ब्यक्तिगत विवरण वा सुचना यो अनुसन्धान गर्ने समुह भन्दा बाहिर कतै खुलाउने छैन?

- ☐ छ
- ☐ छैन

मैले प्रगोग गर शब्द वा भाषालाई प्रकाशन, रिपोर्ट, वेबसाइट वा रिसर्चको अन्य काममा गोप्य रूपमा प्रगोग गरिनेछ?

- ☐ छ
- ☐ छैन

यस सूचना यो अनुसन्धान बाहेक अन्य प्रयोजनको लागि प्रयोग गर्नु परेमा

मैले उपलब्ध गराएको डाटा अभिलेखिकरणमा राख्नको लागि म सहमत छु?

- ☐ छ
- ☐ छैन

मैले दिएको डाटा कृबस वा कृबस भन्दा बाहिरका अन्य अनुसन्धानकर्ताहरुले यस फारम अनुरोध गरे अनुसारकै ब्यक्तिगत जानकारीको गोपनियता सुरक्षित गर्ने भएमा मात्रै डाटाको प्रयोग गर्न पाउनेछ?

- ☐ छ
- ☐ छैन

मैले दिएको डाटा कृबस वा कृबस भन्दा बाहिरका अन्य अनुसन्धानकर्ताहरुले मेरो शब्द वा भाषाहरूलाई प्रकाशन, रिपोर्ट, वेबसाइट वा रिसर्चको अन्य काममा प्रयोग गर्दा गोप्य रुपले प्रयोग गर्नु पर्नेछ।

- ☐ हुन्छ
- ☐ हुदैन

सहभागीको नाम:

हस्ताक्षर:

मिति:
