# Supplementary material for: Development of Data Transfer Ethics Framework (daTEF): A participatory approach to delivering evidence-based guidelines for healthcare data transfer
Source: PLoS One. 2025 Nov 10;20(11):e0336389. doi: 10.1371/journal.pone.0336389 (PMC12599928; doi:10.1371/journal.pone.0336389)
Supplement: S3 File — (PDF) [file pone.0336389.s003.pdf]

Characterizing the challenges experienced in health care data exchange between Nepalese and international research team to develop a local data transfer framework

Participant Information Sheet

Prospective Study- Adult providing own consent.

Title: Characterizing the challenges experienced in health care data exchange between Nepalese and international research team to develop a local data transfer framework

Short Title: daTEF

Protocol Number: 1.0

Project Sponsor: Public Health Alliance for Genomic Epidemiology

Investigators

Principal Investigator**:** Anurag Adhikari (PhD)

Co- Investigator**:** Minu Singh (M.Sc.)

Co- Investigator**:** Gopiram Syangtan (M.Sc.)

Location: Kathmandu Research Institute for Biological Sciences (KRIBS)
Biomedicum Research Campus Building
Saptakhel-9, Balkumari, Chyasal Road,
Lalitpur 44700, Bagmati Province, Nepal

Introduction

You are invited to take part in this study which relies on a participatory approach based on the opinion and discussion forum directly from those who have been engaged in the multinational healthcare research study in Nepal in different managerial and research capacities. This participant information sheet tells you about the research study. It explains the tests and processes involved. Knowing what is involved will help you decide if you want to take part in the study.

Please take your time reading this information. For any topic that you are unclear about or would want additional information on, ask questions about it. You might want to discuss it with a family member, friend, or your team members before deciding whether to participate. This study only accepts voluntary participation. It is not necessary for you to participate if you don't want to.

What does my participation involve?

If you decide, you want to take part in the research study, you will be asked to sign the consent section. By signing it, you are telling us that you:

• Understand what you have read.

• Consent to take part in the research study.

• Consent to Use of the information you provide for this study only.

You will be given a copy of this participant information sheet to keep.

What is the purpose of this research?

The purposes of this research are to develop a framework for addressing systemic and individual challenges observed during the implementation and execution of healthcare research that involves healthcare data transfer outside of Nepal. This study will include interacting with identified study participants to identify the challenges of data transfer and data ownership, development of a draft summary of the challenges and distribution of the draft for comments to the participants, and creation of a final guideline from the data collected to publish as an open-source framework.
This study is under the supervision of principal investigator Anurag Adhikari and co-investigators Minu Singh and Gopiram Syangtan from Kathmandu Research Institute for Biological Sciences (KRIBS).

What does participation in this research involve?

This is a cross-sectional, descriptive qualitative study. Principal Investigator Anurag Adhikari will design and supervise the proposed study activities, reporting, and result dissemination. Co-Investigators Minu Singh and Gopiram Syangtan will do the identification and recruitment of study participants, data analysis, and draft preparation. The team member Sauhardra Manandhar will contribute as a face-to-face session moderator and data collector and analysis. Cross-validation of all the tasks will be done regularly in a blinded fashion by Anurag Adhikari. Confidentiality will be maintained and monitored under the supervision of Co-Principal investigator Gopiram Syangtan.

The requirements of all the participants in this study will be:

- Review the information sheet and if agree sign the consent form.
- Taking part in the study include, filling up the semi-structured questionnaire form, being interviewed, and being recorded.

How will the research be monitored?

Every two weeks, our research team members will monitor the conduct of the research. In addition, staff involved in facilitating the smooth running of the Study development of the data transfer

framework will be in regular communication with the principal researcher regarding the conduct and progress of the study.

Will taking part in this study cost me anything, and will I be paid?

There are no costs associated with the participants participating in this research study.

What do I have to do?

If you decide to participate, we will ask you to fill in the consent form and participate in the scheduled interview date and time, which will be either one by one by a virtual platform like Zoom or by arranging face to face meeting, where you need to answer and provide your experience and suggestion on a set of semi-structured questionnaires prepared for the research study.

What is the frequency of the participant's involvement in the research?

Only once.

Do I have to take part in this research project?

If you do not wish to take part, you do not have to. Your relationship with those assisting you will not be affected in any way if you decide not to take part. If you do decide to take part, you will be given a Consent Form to sign and you will be given a copy of this sheet to keep.

What are the possible benefits of taking part?

We cannot guarantee or promise that you will receive any direct benefits from this study just now; however, potential benefits in the future might be identifying the prevailing challenges and current local solutions in healthcare data transfer from Nepal to international collaborators and vice versa.

Are vulnerable participants involved?

No.

Are there any risks involved for the participants?

No

Confidentiality / Privacy

Under Nepal Health privacy law, all information collected about you must be kept confidential, unless you agree to it being released. If you consent to take part in this study, the data collected for the study will be looked at by the research team. They may also be looked at by authorized people from the research team to check that the study is being carried out correctly. All of these people will have a duty of confidentially to you as a research participant and no information that could identify you will be given to anyone else.

The results of this study will be published, for example in scientific journals, you will not be identified by name and a summary of findings will be provided to you by letter. In addition, any information where potentially identifiable, will not be released for other uses without the participant's prior consent, unless required by law.

What if I withdraw from this research study?

If you wish to withdraw from the study after it has started, you can do so at any time without having to give a reason. There will be no consequences to you for withdrawing from the study.

If you do decide to withdraw from the study, please notify the research team. This notice will allow the research supervisors time to discuss any special requirements linked with the study.

Who is organizing and funding the research?

This research is being funded by the Public Health Alliance for Genomic Epidemiology, University of the Western Cape, Robert Sobukwe Rd, Bellville, Cape Town, 7530, South Africa.

Who has reviewed the research project?

This research is reviewed by Ethical Review Board (ERB) of Nepal Health Research Council (NHRC), Ramshah Path, Kathmandu, Nepal.

Call: +977-01-4254220

Email: nhrc@nhrc.gov.np

Website: https://nhrc.gov.np/ethics/ethical-review-board/

Further information and who to contact.

When you have read this information, the researcher will discuss with you any queries you may have. If you would like to know more at any stage, please do not hesitate to contact the **Kathmandu Research Institute for Biological Sciences**, **Bio-Medicum Research Campus Building**, Saptakhel-9, Balkumari, Chyasal Road, Lalitpur 44700, Nepal

Website: https://kribs.org.np/

Email: info@kribs.org.np

Investigators: - **Anurag Adhikari** (email: adhikari.a@kribs.org.np), **Minu Singh** (email: singh.m@kribs.org.np), **Gopiram Syangtan** (syangtan.g@kribs.org.np)]

Characterizing the challenges experienced in health care data exchange between Nepalese and international research team to develop a local data transfer framework

सहभागी जानकारी पाना

शीर्षक: डाटा ट्रान्सफर फ्रेमवर्क विकास गर्न नेपाली र अन्तर्राष्ट्रिय अनुसन्धान टोलीबीच स्वास्थ्य सेवा डाटा आदानप्रदानमा अनुभव भएका चुनौती चित्रण

छोटो शीर्षक: daTEF

प्रोटोकल नम्बर: १.०

परियोजना प्रायोजक: पब्लिक हेल्थ एलायन्स फर जेनोमिक एपिडेमियोलोजी

अन्वेषकहरू

प्रमुख अन्वेषक: अनुराग अधिकारी (Ph.D)

सह अन्वेषक: मिनु सिंह (M.Sc.)

सह अन्वेषक: गोपीराम स्याङ्तान (M.Sc.)

स्थान: डिपर्ट्मेन्ट अफ इन्फेक्सन एण्ड इम्युनोलोजी काठमाडौं रिसर्च ईन्स्टिच्यु फर बायोलोजीकल साइन्सेस (कृबस)

बायोमेडिकम रिसर्च क्याम्पस भवनसप्तखेल-९,

बालकुमारी, च्यासल रोड

ललितपुर ४४७००, बागमती प्रदेश, नेपाल

परिचय

नेपालमा विभिन्न व्यवस्थापकीय र अनुसन्धान क्षमतामा बहुराष्ट्रिय स्वास्थ्य सेवा अनुसन्धान अध्ययनमा संलग्न व्यक्तिहरूबाट प्रत्यक्ष रूपमा राय र छलफल फोरममा आधारित सहभागितामूलक दृष्टिकोणमा आधारित यस अध्ययनमा भाग लिन तपाईंलाई निमन्त्रणा गरिएको छ। यो सहभागी सूचना पत्रले तपाईंलाई अनुसन्धान अध्ययनको बारेमा बताउँछ। यसले परीक्षणहरू र प्रक्रियाहरू समावेश गर्दछ। के समावेश छ भन्ने कुरा थाहा पाउँदा तपाइँ अध्ययनमा भाग लिन चाहनुहुन्छ कि चाहनुहुन्न भनेर निर्णय गर्न मद्दत गर्नेछ।

कृपया यो जानकारी पढ्न आफ्नो समय लिनुहोस्। कुनै पनि विषय जसको बारेमा तपाई अस्पष्ट हुनुहुन्छ वा थप जानकारी चाहानुहुन्छ, यसको बारेमा प्रश्नहरू सोध्नुहोस्। तपाईं भाग लिने कि नलिने निर्णय गर्नु अघि परिवारको सदस्य, साथी, वा तपाईंको टोलीका सदस्यहरूसँग छलफल गर्न सक्नुहुन्छ। यो अध्ययनले स्वैच्छिक सहभागिता मात्र स्वीकार गर्दछ। यदि तपाइँ चाहनुहुन्न भने तपाइँ सहभागी हुन आवश्यक छैन।

मेरो सहभागितामा के समावेश छ?

यदि तपाईंले अनुसन्धान अध्ययनमा भाग लिने निर्णय गर्नुभयो भने, तपाईंलाई सहमति खण्डमा हस्ताक्षर गर्न भनिनेछ। यसलाई हस्ताक्षर गरेर, तपाईंले हामीलाई भन्नुभएको छ कि तपाईं:

• यस पत्रमा पढेको कुरा बुझ्नुभयो |

• अनुसन्धान अध्ययनमा भाग लिनको लागि सहमति।

• यस अध्ययनको लागि तपाईंले प्रदान गर्नुभएको जानकारी प्रयोग गर्न सहमति।

• यस अध्ययनभन्दा बाहिर तपाईंले प्रदान गर्नुभएको जानकारीको प्रयोग गर्न सहमति।

तपाईलाई यो सहभागी जानकारी पत्रको प्रतिलिपि राख्नको लागि दिइनेछ।

यो अनुसन्धानको उद्देश्य के हो?

यस अनुसन्धानको उद्देश्य भनेको नेपाल बाहिर स्वास्थ्य सेवा डाटा स्थानान्तरण समावेश गर्ने स्वास्थ्य सेवा अनुसन्धानको कार्यान्वयन र कार्यान्वयनको क्रममा देखिएका सिन्डेमिक(एक भन्दा बढी महामारीको सह-घटना) चुनौतीहरूलाई सम्बोधन गरी दिशानिर्देश विकास गर्नु हो , जसमा पहिचान अध्ययन सहभागीहरूसँग अन्तरक्रिया गर्ने समावेश छ। चुनौतीहरूको मस्यौदा सारांशको विकास र सहभागीहरूलाई टिप्पणीहरूको लागि मस्यौदाको वितरण र खुला स्रोत नीति ढाँचाको रूपमा प्रकाशित गर्न सङ्कलन गरिएको डाटाबाट अन्तिम दिशानिर्देश सिर्जना गर्नु हो ।

यो अध्ययन काठमाडौं रिसर्च इन्स्टिच्युट फर बायोलोजिकल साइन्सेस (कृबस) का प्रमुख अन्वेषक अनुराग अधिकारी र सहअन्वेषक मिनु सिंह र गोपीराम स्याङ्तानको निरीक्षणमा भएको हो ।

यस अनुसन्धानमा सहभागिता के समावेश छ?

यो एक क्रस-सेक्शनल, वर्णनात्मक अध्ययन हो। प्रमुख अन्वेषक अनुराग अधिकारीले प्रस्तावित अध्ययन गतिविधि, प्रतिवेदन र परिणाम वितरणको डिजाइन र सुपरिवेक्षण गर्नेछन्। सह-अन्वेषक मिनु सिंहले अध्ययन सहभागीहरूको पहिचान र भर्ती, डाटा विश्लेषण र मस्यौदा तयारी गर्नेछन्। टोलीका सदस्य सौहाद्र मानन्धरले फेस टु फेस अन्तर्वार्ता र तथ्याङ्क सङ्कलन तथा विश्लेषणमा योगदान दिनेछन् । अनुराग अधिकारीद्वारा सबै कार्यको क्रस भ्यालिडेसन नियमित रूपमा गरिनेछ। सह-अन्वेषक गोपीराम स्याङ्तानको निगरानीमा गोपनीयता कायम र अनुगमन गरिनेछ।

यस अध्ययनका सबै सहभागीहरूको आवश्यकताहरू निम्न हुनेछन्:

- जानकारी पत्र समीक्षा गर्नुहोस् र सहमत भएमा सहमति फारममा हस्ताक्षर गर्नुहोस्।
- यो अध्ययनमा भाग लिनुमा अर्ध-संरचित प्रश्नावली फारम भर्नु, अन्तर्वार्ता लिनु र रेकर्ड गर्नु समावेश छ।

अनुसन्धान कसरी अनुगमन हुनेछ?

प्रत्येक दुई हप्तामा प्रमुख अनुसन्धानकर्ताको सुपरभाइजरी प्यानल (प्रमुख अन्वेषक, सह-अन्वेषकहरू र अध्ययनमा संलग्न सबै टोली सदस्यहरू समावेश छन्) निर्धारित बैठकहरूमा अनुसन्धानको आचरणको अनुगमन गर्नेछ। थप रूपमा, डाटा ट्रान्सफर फ्रेमवर्कको अध्ययन विकासको सहज सञ्चालनमा संलग्न कर्मचारीहरूले अध्ययनको आचरण र प्रगतिको बारेमा सिद्धान्त अनुसन्धानकर्तासँग नियमित सञ्चारमा रहनेछन्।

के यस अध्ययनमा भाग लिनको लागि मलाई केही खर्च लाग्छ, र के मलाई भुक्तानी गरिनेछ?

यस अनुसन्धान अध्ययनमा भाग लिन केही खर्च लागनेछैन र कुनै भुक्तानी पनि गरिनेछैन।

मैले के गर्नुपर्छ?

यदि तपाइँ भाग लिने निर्णय गर्नुहुन्छ भने, हामी तपाइँलाई सहमति पत्र भर्न र निर्धारित अन्तर्वार्ता मिति र समय मा भाग लिन अनुरोध गर्नेछौं, जुम जस्तै भर्चुअल प्लेटफर्म द्वारा वा आमनेसामने भेटघाट व्यवस्थित गरेर हुनेछ, जहाँ तपाईंले अनुसन्धान अध्ययनको लागि तयार पारिएको अर्ध-संरचित प्रश्नावलीहरूको सेटमा आफ्नो अनुभव र सुझावको जवाफ दिनु पर्छ।

अनुसन्धानमा सहभागीको संलग्नताको आवृत्ति के हो?

एक पटक मात्र।

के मैले यस अनुसन्धान परियोजनामा भाग लिनुपर्छ?

यदि तपाइँ भाग लिन चाहनुहुन्न भने, भाग लिन आवश्यक छैन। तपाईंले भाग नलिने निर्णय गरे पनि तपाईंलाई सहयोग गर्नेहरूसँगको तपाईंको सम्बन्धमा कुनै पनि हिसाबले असर पर्ने छैन। यदि तपाईंले भाग लिने निर्णय गर्नुभयो भने, तपाईंलाई हस्ताक्षर गर्नको लागि सहमति फारम दिइनेछ र तपाईंलाई राख्नको लागि यो पत्रको प्रतिलिपि दिइनेछ।

भाग लिनुको सम्भावित फाइदाहरू के हुन्?

हामी ग्यारेन्टी वा प्रतिज्ञा गर्न सक्दैनौं कि तपाईंले अहिले नै यस अध्ययनबाट कुनै प्रत्यक्ष लाभहरू प्राप्त गर्नुहुनेछ; यद्यपि, सम्भावित भविष्यका फाइदाहरूमा स्वास्थ्य सेवा अनुसन्धानको कार्यान्वयन र कार्यान्वयनको क्रममा देखिएका विभिन्न सिन्डेमिक चुनौतीहरूलाई सम्बोधन गर्न दिशानिर्देश विकास गर्ने सम्भावना समावेश हुन सक्छ ।

के असुरक्षीत सहभागीहरू संलग्न छन्?

छैन।

के त्यहाँ सहभागीहरूको लागि कुनै जोखिम समावेश छ?

छैन ।

गोपनीयता सम्बन्धमा

नेपाल स्वास्थ्य गोपनीयता कानून अन्तर्गत, तपाइँ बारे संकलित सबै जानकारी गोप्य राख्नुपर्छ, जब सम्म तपाइँ यसलाई जारी गर्न सहमत हुनुहुन्न। यदि तपाइँ यस अध्ययनमा भाग लिन सहमत हुनुहुन्छ भने, अध्ययनको लागि सङ्कलन गरिएको डाटा अनुसन्धान टोलीद्वारा हेरिनेछ। उनीहरूलाई अनुसन्धान टोलीका अधिकृत व्यक्तिहरूले पनि अध्ययन सही रूपमा भइरहेको छ कि छैन भनेर जाँच गर्न सकिन्छ। यी सबै व्यक्तिहरूको गोपनीयता एक अनुसन्धान सहभागीको रूपमा गोप्य राख्नु तपाइँको कर्तव्य हुनेछ र तपाइँलाई पहिचान गर्न सक्ने कुनै पनि जानकारी अरू कसैलाई दिइने छैन।

यस अध्ययनका नतिजाहरू प्रकाशित गरिनेछन्, उदाहरणका लागि वैज्ञानिक पत्रिकाहरूमा, तपाईंलाई नामद्वारा पहिचान गरिने छैन र निष्कर्षहरूको सारांश तपाईंलाई पत्रद्वारा उपलब्ध गराइनेछ। थप, कानून द्वारा आवश्यक नभएसम्म कुनै पनि जानकारी (जहाँ सम्भावित रूपमा पहिचान गर्न सकिन्छ) सहभागीको पूर्व सहमति बिना अन्य प्रयोगहरूको लागि जारी गरिने छैन,

के हुन्छ यदि म यो अनुसन्धान अध्ययनबाट पछि हट्छु?

यदि तपाइँ यो अध्ययन सुरु गरे पछि फिर्ता हुन चाहनुहुन्छ भने, तपाइँ कुनै कारण नदिई कुनै पनि समयमा त्यसो गर्न सक्नुहुन्छ। अध्ययनबाट पछि हट्दा तपाईलाई कुनै असर हुनेछैन।

यदि तपाईंले अध्ययनबाट फिर्ता हुने निर्णय गर्नुभयो भने, कृपया अनुसन्धान टोलीलाई सूचित गर्नुहोस्। यस सूचनाले अनुसन्धान पर्यवेक्षकहरूलाई अध्ययनसँग सम्बन्धित कुनै पनि विशेष आवश्यकताहरू छलफल गर्न समय दिनेछ।

अनुसन्धानको आयोजना र आर्थिक सहयोग कसले गरिरहेको छ?

यो अनुसन्धानको आयोजना र आर्थिक सहयोग पब्लिक हेल्थ एलायन्स फर जेनोमिक एपिडेमियोलोजी, वेस्टर्न केप विश्वविद्यालय, रोबर्ट सोबुक्वे आरडी, बेलभिल, केप टाउन, 7530, दक्षिण अफ्रिका द्वारा भइरहेको छ।

अनुसन्धान परियोजनाको समीक्षा कसले गरेको छ?

यो अनुसन्धान नैतिक समीक्षा बोर्ड (ERB) नेपाल स्वास्थ्य अनुसन्धान परिषद् (NHRC) रामशाह पथ काठमाडौं नेपाल द्वारा समीक्षा गरिएको हो।

फोन: +977-01-4254220

इमेल: nhrc@nhrc.gov.np

वेबसाइट: https://nhrc.gov.np/ethics/ethical-review-board/

थप जानकारी र कसलाई सम्पर्क गर्ने

जब तपाईंले यो जानकारी पढ्नुभयो पढीसक्नुहुन्छ, अनुसन्धानकर्ताले तपाइँसँग हुन सक्ने कुनै पनि प्रश्नहरू छलफल गर्नेछन्। यदि तपाईं कुनै पनि चरणमा थप जान्न चाहनुहुन्छ भने, कृपया काठमाडौं अनुसन्धान संस्थान जैविक विज्ञान, सप्तखेल-९, बालकुमारी, च्यासल रोड, ललितपुर ४४७००, नेपालमा सम्पर्क गर्न नहिचकिचाउनुहोस्।

अन्वेषकहरू:- **अनुराग अधिकारी** (इमेल: adhikari.a@kribs.org.np) , **मिनु सिंह** (इमेल: singh.m@kribs.org.np), **गोपीराम स्याङ्तान** (syangtan.g@kribs.org.np)

इमेल: info@kribs.org.np

वेबसाइट: https://kribs.org.np/
