# Supplementary material for: Development of Data Transfer Ethics Framework (daTEF): A participatory approach to delivering evidence-based guidelines for healthcare data transfer
Source: PLoS One. 2025 Nov 10;20(11):e0336389. doi: 10.1371/journal.pone.0336389 (PMC12599928; doi:10.1371/journal.pone.0336389)
Supplement: S4 File — (PDF) [file pone.0336389.s004.pdf]

Characterizing the challenges experienced in health care data exchange between Nepalese and international research team to develop a local data transfer framework

Questionnaires Form (daTEF)

* Required

Name of participants (सहभागीको नाम)*:

Profession (पेशा):

Email*(इमेल) *:

Phone/Mobile No. (फोन/मोबाइल नम्बर):

Organization (संस्था)*:

1. Have you worked with an international Organization/company/Institute? *(Mark only one)

के तपाईंले अन्तर्राष्ट्रिय संस्था/कम्पनीसँग काम गर्नुभएको छ? *(एक मात्र चिन्ह लगाउनुहोस्।)

☐ Yes (छ)

☐ No (छैन)

If you have chosen yes above (यदि तपाईंले माथि छ भन्ने रोज्नुभएको छ भने)

2. What are the types of international organization you have collaborated with yet? *

(तपाईंले अहिलेसम्म कस्ता प्रकारका अन्तर्राष्ट्रिय संस्थाहरूसँग सहकार्य गर्नुभएको छ? *)

☐ Government Organization (सरकारी संस्था)

☐ Non-government Organization (गैर सरकारी संस्था)

☐ Academic institute/University (शैक्षिक संस्थान/विश्वविद्यालय)

☐ Research Centre/Institute (अनुसन्धान केन्द्र/संस्था)

☐ Industry (उद्योगहरू)

☐ International Agencies (अन्तर्राष्ट्रिय एजेन्सीहरू) (WHO, UNICEF, UN)

☐ More than one above (माथि एक भन्दा बढी)

3. How many collaborative organizations have you work until today? *

(तपाईंले आज सम्म त्यस्तो कतिवटा संस्थाहरूसँग काम गर्नुभयो?) *

☐ 1-2 (१-२)

☐ 3-4 (३-४)

☐ 5-10 (५-१०)

☐ >10 (> १०)

4. How often do you interact/meeting with your collaborators currently or in past? *
(तपाईंले हाल वा विगतमा आफ्ना सहकार्य संस्थाको सहकर्मीहरूसँग कति पटक अन्तरक्रिया/भेट गर्नुहुन्छ?) *

☐ Once in a week (हप्तामा एक पटक)

☐ Once in a month (महिनामा एक पटक)

☐ Three times in a year (एक वर्षमा तीन पटक)

☐ Twice in a year (एक वर्षमा दुई पटक)

☐ Once in a year (वर्षमा एक पटक)

5. Did you reach an agreement on data transfer schematics before you began working with your collaborators? *
(के तपाईंले आफ्ना सहकार्य संस्थाको सहकर्मीहरूसँग काम सुरु गर्नु अघि डेटा स्थानान्तरण योजनामा सम्झौतामा गर्नुभएको छ?) *

☐ Yes (छ)

☐ No (छैन)

If yes, how did you reach an agreement? (यदि छ भने, तपाईंले कसरी सम्झौता गर्नुभयो?)

☐ By face-to-face talk/conversation series
(आमनेसामने कुराकानी/वार्तालाप श्रृंखला द्वारा)

☐ By virtual meeting (भर्चुअल बैठक द्वारा)

☐ By electronic mail (इलेक्ट्रोनिक मेल द्वारा)

☐ By Send a draft (proper documentation) (कागजात पठाएर (उचित कागजात)

6. Did you share the any specimens and data during the collaborative work? *
(के तपाईंले सहयोगी कार्यको क्रममा कुनै परिक्षणको लागि नमूना र डेटा स्थानान्तरण गर्नुभएको छ?) *

☐ Yes (छ)

☐ No (छैन)

7. How do you share the data with your collaborators? *
(तपाईं आफ्ना सहकार्य संस्थाको सहकर्मीहरूसँग डाटा स्थानान्तरण कसरी गर्नुहुन्छ?) *

☑ By electronic access (इलेक्ट्रोनिक पहुँच द्वारा)

☐ Hardcopy recorded files (रेकर्ड गरिएका हार्डकपी फाइलहरू)

☐ Extracted data only (विश्लेषण गरिएका डाटा मात्र)

☐ Raw data (कच्चा डाटा)

8. What kinds of problem did you face during sharing of the data or specimens? *

(डाटा वा नमूनाहरू साझेदारी गर्दा तपाईंले के कस्तो प्रकारको समस्या सामना गर्नुभयो?) *

☐ Loss of data or specimens (डाटा वा नमूनाहरूको हानि)

☐ Administrative work (Paper work) (प्रशासनिक काम (कागज कार्य)

☐ Privacy of data (डाटाको गोपनीयता सुनिश्चित)

☐ Ethical issue (नैतिक मुद्दा)

☐ Other including publication complications (प्रकाशन जटिलताहरू सहित अन्य)

9. How did you solve the conflict if it is arising? *

(डाटा वा नमूनाहरू साझेदारी गर्दा उब्जिएका समस्या तपाईंले कसरी समाधान गर्नुभयो?) *

☐ End the relation and work (सम्बन्ध र कामको अन्त्य गरेर)

☐ Legal action (कानूनी कारबाहीको प्रक्रियाबाट)

☐ Other (अन्य) ....

10. Would you say that collaboration is necessary for your field due to inadequate equipment and resources?' *

(अपर्याप्त उपकरण र श्रोतसाधनको कारणले तपाईं आफ्नो क्षेत्रको लागि सहयोग आवश्यक छ भनी के तपाईं भन्न सक्नुहुन्छ?) *

☐ Yes (सक्छु)

☐ No (सक्दिन)

☐ Other (अन्य)..........................

11. Prior collaboration experience: "How have you deal with the following aspects of previous collaboration? *

(अघिल्लो सहयोग अनुभवमा तपाईंले अघिल्लो सहयोगी संस्थाहरु संग कुन कुन पक्षमा कसरी काम गर्नुभएको छ?) *

☐ Group division of Labor (श्रमको समूह विभाजन)

☐ Research work coordination (अनुसन्धान कार्य समन्वय)

☐ Research group composition (अनुसन्धान समूह संरचना)

☐ Authorship attribution (लेखकत्व विशेषता)

☐ Other (अन्य)...................................

Open ended question for interview:

12. Can you please describe your role and involvement in multinational healthcare/Life science research studies in Nepal?
 तपाईंले नेपालमा बहुराष्ट्रिय स्वास्थ्य सेवा / जीव बिज्ञानको अध्ययन अनुसन्धानमा आफ्नो भूमिका र संलग्नताको वर्णन गर्न सक्नुहुन्छ?

13. What are the specific challenges you have encountered in the process of exchanging health care/ life science data between Nepalese and international research teams?

नेपाली र अन्तर्राष्ट्रिय अनुसन्धान टोलीहरू बीच स्वास्थ्य सेवा / जीव बिज्ञानको डेटा आदानप्रदान गर्ने प्रक्रियामा तपाईंले सामना गर्नुभएका विशेष चुनौतीहरू के के छन्?

14. In your experience, what are the main issues related to data ownership and patents that arise during the data exchange process?

तपाईको अनुभवमा, डाटा आदानप्रदान प्रक्रियाको क्रममा उत्पन्न हुने डाटा स्वामित्व र पेटेन्टसँग सम्बन्धित मुख्य मुद्दाहरू के के हुन्?

15. Can you discuss any challenges you have faced in terms of future utilization and publication rights of the data collected in multinational healthcare/ life science research studies?

बहुराष्ट्रिय स्वास्थ्य सेवा / जीव बिज्ञानको अध्ययन अनुसन्धानहरूमा सङ्कलन गरिएका डाटाको भविष्यमा उपयोग र प्रकाशन अधिकारको सन्दर्भमा तपाईंले सामना गर्नुभएका कुनै चुनौतीहरूबारे छलफल गर्न सक्नुहुन्छ?

16. How have policies and regulations in Nepal impacted the context of data transfer and sharing in healthcare/ life science research?

नेपालमा नीति र नियमहरूले स्वास्थ्य सेवा / जीव बिज्ञानको अनुसन्धानमा डाटा ट्रान्सफर र साझेदारीको सन्दर्भमा कसरी प्रभाव पारेको छ?

17. What are the decision-making processes within your institution regarding data transfer and how do they contribute to the challenges faced?

डाटा स्थानान्तरण सम्बन्धी तपाइँको संस्थामा निर्णय लिने प्रक्रियाहरू के के हुन् र तिनीहरूले सामना गर्ने चुनौतीहरूमा कसरी योगदान गर्छन्?

18. Have you encountered any specific demands or criteria from third-party funders or organizations involved in the research that have affected data sharing? If so, what were they and how did they impact the process?

के तपाईंले दातृ-निकाय वा अनुसन्धानमा संलग्न संस्थाहरूबाट (तेस्रो-पक्षबाट) डेटा साझेदारीलाई असर गर्ने कुनै विशेष माग वा मापदण्डहरूको सामना गर्नुभएको छ? यदि छ भने, तिनीहरू के थिए र तिनीहरूले साझेदारी प्रक्रियालाई कसरी प्रभाव परेको थियो?

19. What are the knowledge, attitude, and actions of Nepalese researchers towards data sharing, and how do they contribute to the challenges faced in data exchange?

डाटा आदानप्रदानप्रति नेपाली अनुसन्धानकर्ताहरूको ज्ञान, दृष्टिकोण र कार्यहरू के के छन् र उनीहरूले डाटा आदानप्रदानमा सामना गर्नुपर्ने चुनौतीहरूलाई कसरी योगदान पुऱ्याउँछन्?

20. Are there any specific contextual challenges related to policies and regulations in Nepal that impact data transfer in healthcare/ life science research?

नेपालमा नीति र नियमहरूसँग सम्बन्धित स्वास्थ्य सेवा/ जीव बिज्ञानको अनुसन्धानमा डाटा ट्रान्सफरलाई असर गर्ने कुनै खास सन्दर्भगत चुनौतीहरू छन्?

21. What do you expect the government to do in terms of policy to ensure smooth collaborations? *

(सुमधुर सहकार्य सुनिश्चित गर्नको लागि सरकार सँग कस्तो नीतिको अपेक्षा गर्नुहुन्छ?)*

22. Do you believe that scientists should not only publish but also interpret research findings and collaborate with international organizations to inform policymakers? Why? *

(वैज्ञानिकहरूले नीति निर्माताहरूलाई सूचित गर्नका लागि वैज्ञानिकहरूले अनुसन्धान निष्कर्षहरू प्रकाशित मात्र होइन व्याख्या गर्न र अन्तर्राष्ट्रिय संस्थाहरूसँग सहकार्य गर्नुपर्छ भन्ने के तपाईं विश्वास गर्नुहुन्छ? किन?) *

23. Based on your experience, what potential local solutions or work procedures do you suggest to address the challenges identified in data transfer and ownership?

तपाईको अनुभवको आधारमा, डेटा स्थानान्तरण र स्वामित्वमा पहिचान गरिएका चुनौतिहरूलाई सम्बोधन गर्न तपाई कुन कुन सम्भावित स्थानीय समाधान वा कार्यहरू सुझाव दिनुहुन्छ?
