# Supplementary material for: Development of Data Transfer Ethics Framework (daTEF): A participatory approach to delivering evidence-based guidelines for healthcare data transfer
Source: PLoS One. 2025 Nov 10;20(11):e0336389. doi: 10.1371/journal.pone.0336389 (PMC12599928; doi:10.1371/journal.pone.0336389)
Supplement: Attachment — Submitted filename: Authors response_PONE-D-25-14540.pdf [file pone.0336389.s005.pdf]

18 July 2025

To,

The Editor-in-Chief and Academic Editor,
PLOS ONE, San Francisco, CA 94103, USA.

Subject: Responses to Reviewers' Comments

Manuscript: Development of Data Transfer Ethics Framework (daTEF): A participatory approach to delivering evidence-based guidelines for healthcare data transfer (PONE-D-25-14540)

Dear Dr. Kanchan Thapa,

We would like to thank the editor and independent reviewers for dedicating their valuable time to read this manuscript, offering comments and suggestions to enhance the quality of our work. We appreciate the reviewers for providing us with the opportunity to revise and improve our manuscript. All reviewer comments are reproduced below in a table, with corresponding point-by-point responses following each comment, and changes highlighted in blue. The line and page numbers referenced are based on the track-changed version of the revised manuscript.

S.N.	Points/comments to be addressed	Explanation
	**Reviewer 1**	
1.	This manuscript presents a study on ethical data transfer practices, with particular relevance for LMICs navigating research collaborations. The development of a Data Transfer Ethics Framework is a notable contribution, and the study is clearly written, methodologically appropriate, and well-structured.	We (all authors) would like to thank you for your feedback.
2.	My main comment is that I thought Delphi methodology involved repeated interviews to reach consensus, and that this process, if adopted, is not clearly articulated in the manuscript. The link between findings and the final framework could be more explicit.	We have revised Figure 1 and replaced the 'modified Delphi method' with 'a pre-structured survey' throughout the revised manuscript, as advised by reviewer 3.
	**Reviewer 2**	
1.	The paper is well written, considering critical aspects in research, including gaps identified in the literature review to understand potential issues in data transfer and the possible solutions. The study obtained ethics approval from the relevant board. The result synthesis into key thematic areas also makes it easier for readers to understand key findings, as well as the table summary of challenges experienced by scientific researchers in data sharing and the possible solutions.	We (all authors) would like to thank you for your feedback.
2.	The study could improve by addressing some of the possible study limitations as well as providing generalization limits. For example, whether the study is only applicable to Nepal or can be generalized to a larger population (which should be specified).	The revised manuscript includes the 'limitations of this study' (page 13, L316-320).
	Reviewer 3	
1.	The authors have identified four principal challenges in the data transfer process and provided potential solutions. The idea and the findings hold considerable value for the Nepalese research community.	We (all authors) would like to thank you for your feedback.
2.	The authors outlined two stages of the study based on methods and Figure 1:<br>1. The initial 'scoping' stage involved inviting executives and directors from 11 organisations to participate in a workshop with the	We have revised Figure 1 and replaced the 'modified Delphi method' with 'a

	investigator team to assess the research question and study design and to provide suggestions based on their expert evaluation.<br>2. The second 'Delphi' stage involved experts nominated by the panel from the first stage, who underwent a single round of anonymous interviews, questionnaires, and controlled feedback against the identified pillars.<br><br>The authors labelled the study as a modified Delphi technique; however, they reported undertaking only one round of blind interviews and questionnaires. The fundamental structure of the Delphi technique and its variants involves two or more rounds of questionnaires (or the option to respond at least twice) with controlled feedback presented starting at the second round, according to the guidelines.<br><br>The authors reported in lines 107-108 that: 'The Delphi stage utilized a structured and iterative research technique, etc.' The term 'iterative' indicates multiple rounds, which contradicts the description of a single round in lines 109-111: 'The methods involved one round of blind interviews, etc.'<br><br>The statements are contradictory; if the study involved a single round of expert opinions, the characteristics of the study more resemble an expert opinion survey or a structured survey rather than a Delphi survey due to the lack of the iterative nature of the Delphi technique. The methods section needs clarification and subsequent revision of the manuscript	pre-structured survey' throughout the revised manuscript, as advised."
3.	The authors did not highlight any limitations to the study and whether these limitations were based on the study participants (experts gathered), the actual study methods, or the generalizability of the findings.	The revised manuscript includes the 'limitations of this study' (page 13, L316-320).

Thank you so much,

Best regards,

Dr Binod Rayamajhee, on behalf of all authors
UNSW, Sydney, Australia
b.rayamajhee@unsw.edu.au, binod@kribs.org.np
