# Supplementary material for: Development of Data Transfer Ethics Framework (daTEF): A participatory approach to delivering evidence-based guidelines for healthcare data transfer
Source: PLoS One. 2025 Nov 10;20(11):e0336389. doi: 10.1371/journal.pone.0336389 (PMC12599928; doi:10.1371/journal.pone.0336389)
Supplement: Attachment — Submitted filename: Authors response_PONE-D-25-14540TR1_R2.pdf [file pone.0336389.s006.pdf]

26 August 2025

To,

The Editor-in-Chief and Academic Editor,
PLOS ONE, San Francisco, CA 94103, USA.

Subject: Responses to Editor comments

Manuscript: Development of Data Transfer Ethics Framework (daTEF): A participatory approach to delivering evidence-based guidelines for healthcare data transfer (PONE-D-25-14540)

Dear Dr. Kanchan Thapa,

We would like to thank you for dedicating your valuable time to reviewing and editing this manuscript, offering comments and suggestions to enhance the quality of our work. We greatly appreciate your valuable comments and suggestions. All comments are reproduced in the table below, with corresponding point-by-point responses provided for each. Changes have been highlighted in blue, and the referenced line and page numbers correspond to the tracked-changes version of the revised manuscript.

S.N.	Points/comments	Response
1.	I suggest rewriting the ethical consideration section. You added a line about Helsinki declaration, I suggest rewriting the ethical consideration in original language what you exactly did.	We added following details in the ethic section:<br><br>All participants were provided with a Participant Information Sheets (PIS) and gave written informed consent prior to participation. After reviewing the PIS and agreeing to take part as volunteers, participants were interviewed using a pre-structured questionnaire to record their responses.<br><br>Page 6, L145-150.
2.	I suggest modifying your Abstract and background section adding - What is the global significance of this study? Why people outside of country need to read your paper and what they can replicate or modify in their context?	We added the aim of the study and its significance in the current context to the background section of the abstract.<br><br>This study aims to develop a locally governed framework for ethical healthcare data exchange, which is essential for advancing global disease surveillance, strengthening outbreak response, optimizing patient care through comparative best practices, and safeguarding individual privacy and rights.<br><br>Page 1, L17-20.
3.	Results<br>Table 1. Is your table complete? Please review other paper and presenting your table heading an appropriate way.<br><br>Figure2. Is this your table heading?<br>Figure 3: ???	We edited the heading to table 1 as "Types and numbers of invited stakeholders and respondents in the structured questionnaire survey stage". Page 6, L156-157.<br><br>**Figure 2:** Fig. 2 is a map showing the location/address of research institutes enrolled for this study. (Fig. 2 legend is mentioned in Page 6, L152-157)<br><br>**Figure 3:** Fig. 3 is showing the distribution of study participants by professional background. (Fig. 3 legend is mentioned in Page 6, L163).

4.	I suggest to present socio-demographic findings in one table unless and until they really impact on the overall study findings.	Thank you for the advice.<br><br>Given the small sample size, we briefly described the participants' professional experience and background in a paragraph (page 6, L158–162), which is complemented by Figure 3.
5.	Line 174: Despite limited knowledge of data transfer, most respondents were involved in data transfer?<br><br>Please write an appropriate section sub-heading. I think such information as of line 174 should go in the discussions section.	Thank you for the advice.<br><br>A new subheading, 'Participants' experiences and practices related to data sharing,' has been added to the revised manuscript, and the corresponding information has also been incorporated into the Discussion section (page 12, L274–276).
6.	Thank you for adding the limitation section, but I think presenting your strength is equally important.	Thank you for the advice.<br><br>We added 'To our knowledge, it is the first to systematically examine data transfer practices and challenges in Nepal's research context, engaging participants with direct experience in multinational collaborations. By employing a participatory approach, the study provides valuable insights into local practices, knowledge gaps, and barriers, which can inform the development of more ethically and locally grounded data-sharing frameworks in resource-limited settings' in the limitations of the study section of the revised manuscript (page 13, L316-320).

Thank you so much,

Sincerely Yours,

On behalf of all authors,

Binod Rayamajhee, MSc, PhD
UNSW, Sydney, Australia
Kathmandu Research Institute for Biological Sciences (KRIBS), Nepal
rayamajheebinod@gmail.com, binod@kribs.org.np
