# Supplementary material for: Development of Data Transfer Ethics Framework (daTEF): A participatory approach to delivering evidence-based guidelines for healthcare data transfer
Source: PLoS One. 2025 Nov 10;20(11):e0336389. doi: 10.1371/journal.pone.0336389 (PMC12599928; doi:10.1371/journal.pone.0336389)
Supplement: Attachment — Submitted filename: Authors response_PONE-D-25-14540TR2.pdf [file pone.0336389.s008.pdf]

06 October 2025

To,

The Editor-in-Chief and Academic Editor,
PLOS ONE, San Francisco, CA 94103, USA.

Subject: Responses to Editor comments

Manuscript: Development of Data Transfer Ethics Framework (daTEF): A participatory approach to delivering evidence-based guidelines for healthcare data transfer (PONE-D-25-14540)

Dear Dr. Kanchan Thapa,

We would like to thank you for dedicating your valuable time to reviewing and editing this manuscript, offering comments and suggestions to enhance the quality of our work. We greatly appreciate your valuable comments and suggestions. All comments are reproduced in the table below, with corresponding point-by-point responses provided for each. Changes have been highlighted in blue, and the referenced line and page numbers correspond to the tracked-changes version of the revised manuscript.

S.N.	Points/comments	Response
**Reviewer #4**		
1.	Abstract:<br>• For methods, dates of the interview can be left out since they appear in the main methods section.<br>• For results, can you summarize these cutting out explanations as you have given.<br>• The abstract should be summarized further; it is very long.	Thank you for your advice<br><br>We have reduced texts in the abstract section.
2.	Methods:<br>• What sampling method did you use?<br>• Can we have more about the inclusion criteria for the study participants? For example, how long should they have stayed in the study area? Or did you take on even those who had recently relocated to your study area?<br>• No pretest of the data collection instrument has been reported.<br>• How did you take care of anonymity and confidentiality? For example, were	Random sampling method was used for the selection of respondents from the invited institutes (L 117, pg 4/16).<br><br>Inclusion criteria of respondents were "having co-authored at least one scientific paper and/or engaged in a co-project with an international partner in the last five years," mentioned in the methodology sections (L 133-134, pg 5/16)<br><br>During the preliminary scoping stage, a small group of experts provided suggestions for the questions, and modifications were made to the pre-structured questionnaire (L 113-121, pg 4/16).

	the interviews in enclosed or open but private spaces? Where these chosen by the participants or the interviewer?	All Interviews were recorded through Zoom (version 5.10.1, 4420). Subsequently, each recording was transferred to the project computer in the form of an audio file. Following the deidentification process of these audio files, manual transcription was carried out. The original file, containing the audio recordings, was securely stored on the project computer, protected by encryption and a password.<br>These details are mentioned in the "Data Management and Translation" section of methodology section (L 143-146, pg 5/16).
3.	Conclusion: This seems so thin. Of what importance are the study findings regionally and internationally, not only in Nepal? Let this clearly stand out because it is the gist of what your study is adding to the field of research.	We have revised the conclusion section in the revised manuscript (L329-339, pg 13/16).
**Reviewer #5**		
1.	Abstract<br>• (L22, L93): The manuscript describes the study design inconsistently, referring to it as both a cross-sectional qualitative study (L 22) and a mixed-methods approach (L 93). Please clearly define the study design and ensure consistency across the abstract and methods.<br>• (L18-20, L 86-88): Aims differ between the abstract and introduction sections; one aims to develop a framework for ethical healthcare data transfer, while the other seeks to present recommendations based on reported local challenges. Please revise the objectives to reflect the actual contribution.<br>• The abstract claims the framework is "essential for advancing global disease surveillance, strengthening outbreak response, optimizing patient care, and safeguarding privacy." Yet, the introduction and discussion did not explain how these global impacts would be achieved. Please either expand on these links or adjust the claim to match the study's scope.	We have edited the abstract in the revised manuscript (l 15-51, pg 1-2/16).
2.	Introduction:<br>Line 75-78: Please provide references or specify details to substantiate these claims.	Reference (ref. no. 12) has been cited in the revised manuscript (L 91, pg 3/16).
3.	Figure 3, 5 (A, B): Current figures are difficult to interpret without percentages.	We have added numerical values (%) in the revised figures 3 and 5.

	Please provide percentages in the charts or present the data in tables.	
4.	L175–176: Figure mislabeled, text refers to 5B but marked as 5A.	We have edited in the revised manuscript (L 191-197, pg 7/16).
5.	L180–181: Statement does not match the type of data sharing shown in Figure 5B.	We have edited in the revised manuscript (L 197, pg 7/16).
6.	L221: The heading should specify biological sample transfer.	We have edited in the revised manuscript (L 244, pg 10/16).
7.	L248–250: The text refers to the need for "well-defined agreements," but it does not explain what constitutes such an agreement or what criteria should be included. Please clarify.	Well-defined agreement refers to a clearly defined the details of data ownership and rights over shared research data in a material transfer agreement (MTA) and Memorandum of Understanding (MoU) before the start of the study between collaborating parties.<br>This is added in the revised manuscript (L 272-276, pg 11/16).

Thank you so much,

Sincerely yours,

On behalf of all authors,

Binod Rayamajhee, MSc, PhD
UNSW, Sydney, Australia
Kathmandu Research Institute for Biological Sciences (KRIBS), Nepal
rayamajheebinod@gmail.com, binod@kribs.org.np
