# Supplementary material for: Development of Data Transfer Ethics Framework (daTEF): A participatory approach to delivering evidence-based guidelines for healthcare data transfer
Source: PLoS One. 2025 Nov 10;20(11):e0336389. doi: 10.1371/journal.pone.0336389 (PMC12599928; doi:10.1371/journal.pone.0336389)
Supplement: Attachment — Submitted filename: Review_daTEF_Nepal_Sept16.docx [file pone.0336389.s007.docx]

**Development of Data Transfer Ethics Framework (daTEF): A participatory approach to delivering evidence-based guidelines for healthcare data transfer**

Binod Rayamajhee, PhD UNSW Medicine & Health: University of New South Wales Medicine & Health Kensington, NSW AUSTRALIA

Gopiram Syangtan, M.Sc.

Sauhardra Manandhar, B. Tech in Biotechnology

Manjula Bhattarai, M.Sc.

Binu Shah, M.Sc Minu Singh, M.Sc.

Binod Rayamajhee, PhD

Anurag Adhikari, PhD

Thank you for submitting your manuscript.

**Abstract**

1. For methods, dates of the interview can be left out since they appear in the main Methods section.
2. For Results, can you summarize these cutting out explanations as you have given.
3. The Abstract should be summarized further, it is very long.

**Introduction**

What is the rate of unmet contraceptive use in Africa, East Africa (if these are documented) before you scale it down to Rwanda? A comparison is important for readers to know how critical the situation.

**Methods**

1. What sampling method did you use?
2. Can we have more about the inclusion criteria for the study participants? For example, how long should they have stayed in the study area? Or did you take on even those who had recently relocated to your study area?
3. No pretest of data collection instrument has been reported.
4. How did you take care of anonymity and confidentiality? For example, were the interviews in enclosed or open but private spaces? Where these chosen by the participants or the interviewer?

**Results**

Good.

**Discussion**

Good.

**Conclusion**

This seems so thin. Of what importance are the study findings regionally and internationally, not only in Nepal? Let this clearly stand out because it is the gist of what your study is adding to the field of research.

**These days women decide”: contraceptive decision-making among IUD users in Uganda:
A qualitative study.**

Authors:

1. **Emelie Looft-Trägårdh**, *Corresponding author*

Department of Women and Children’s Health, Karolinska Institutet

2. **Herbert Kayiga**

Department of Obstetrics and Gynecology, Makerere University College of Health Sciences, P.O. **Othman Kakaire**

Department of Obstetrics and Gynecology, Makerere University College of Health Sciences

3. **Josaphat Byamugisha**

Department of Obstetrics and Gynecology, Makerere University College of Health Sciences

4. **Kristina Gemzell-Danielsson**

Department of Women and Children’s Health, Karolinska Institutet

5. **Amanda Cleeve**

Department of Women and Children’s Health, Karolinska Institutet

**Background**

What is the global situation like before you scale it down to middle and low-income countries and Uganda?

**Methods**

Line 136: Since you name Kawempe hospital, isn’t it better to name the other healthcare facilities too?

Line 143: Were the interviews individual or group?

How did you take care of anonymity and confidentiality? For example, was the interview in an enclosed space or open but private place?

**Data Analysis in 2 sections?**

Which NVivo software version was used?

**Acknowledgements**

Line 596: Luganda not Lugandan

1. Thank you for sending in your manuscript
   Are there any prevalence rates for under nutrition in Uganda and northern or Karamoja region, if any? What do health reports such as UBOS/UDHS or World Bank say? Can these be compared to rates at levels of east Africa, Africa or globally?
2. Line 152: I think an inclusion criterion of how long participants stayed in the area was left out. Or did you take on even those who had recently relocated to your study area?
3. No pretest of data collection instrument has been reported.
4. Rigor on how the FGDs were done should be given. For example, did you give participants cards with random numbers which they would flash whenever they had to talk? That way, documenting their views according to card numbers would easy.

Thank you for sending in your manuscript.

**Background**

Line 51, can you write WHO in full before it is abbreviated the first time. Same edits for other abbreviations for example on line 65 and 67.

**Methods**

6. No mention of where and how the pretest of data collection instrument was done.
7. Rigor on how the FGDs were done should be given. For example, did you give participants cards with random numbers which they would flash whenever they had to talk? That way, documenting their views according to card numbers would be easier yet anonymous.
